# Automatic monitoring of single-wall MAPSE by transesophageal echocardiography for tracking global left ventricular function irrespective of regional hypokinesia: a secondary analysis

**DOI:** 10.1186/s40635-026-00898-1

**Published:** 2026-04-14

**Authors:** Jinyang Yu, Ola Grude, Erik Andreas Rye Berg, Anders Austlid Taskén, Tomas Dybos Tannvik, Katrine Hordnes Slagsvold, Idar Kirkeby-Garstad, Bjørnar Grenne, Svend Aakhus, Gabriel Kiss

**Affiliations:** 1 Department of Circulation and Medical Imaging, Norwegian University of Science and Technology, Trondheim, Norway; 2 Department of Anesthesia and Intensive Care, St. Olav’s Hospital, Trondheim University Hospital, Trondheim, Norway; 3 Clinic of Cardiology, St. Olav’s Hospital, Trondheim University Hospital, Trondheim, Norway; 4 Department of Computer Science, Norwegian University of Science and Technology, Trondheim, Norway; 5 Clinic of Cardiothoracic Surgery, St. Olav’s Hospital, Trondheim University Hospital, Trondheim, Norway

**Keywords:** MAPSE, Left ventricular function, Transesophageal echocardiography, Deep learning, Artificial intelligence, Hemodynamic monitoring, Regional wall motion abnormalities

## Abstract

**Background:**

Measuring mitral annular plane systolic excursion (MAPSE) serially in a single wall may be an effective method for monitoring global left ventricular (LV) function, especially when automated with a novel deep learning method using transesophageal echocardiography, called *autoMAPSE*. However, this assumption is untested in postoperative ICU patients, and the impact of regional wall motion abnormalities (RWMA) is unclear.

**Aim:**

To assess the ability of single-wall autoMAPSE in tracking changes in global LV function (i.e., trending ability) using manual global MAPSE averaged from four walls as reference, and to explore how this ability was affected by RWMA.

**Methods:**

This study was a secondary analysis of a prospective observational study. The changes in single-wall autoMAPSE and manual global MAPSE were calculated in 49 patients undergoing cardiac surgery. *Trending ability* evaluates how well a novel method detects changes in a reference method, which we assessed using four-quadrant plots with concordance rates. To explore the effects of RWMA, we classified RWMA by temporal behavior (persistent or dynamic) and by location relative to single-wall autoMAPSE (same or remote wall), yielding four patterns for analysis of trending ability: (i) remote wall, dynamic RWMA, (ii) remote wall, persistent RWMA, (iii) same wall, dynamic RWMA, and (iv) same wall, persistent RWMA.

**Results:**

Overall, single-wall autoMAPSE had adequate trending ability (concordance rate 91%). All LV walls showed adequate trending ability (concordance rate ≥ 90%), except for the septal wall (concordance rate 88%). The effect of RWMA was negligible, as the concordance rates of the four patterns ranged from 89 to 96%.

**Conclusions:**

In this secondary analysis, single-wall autoMAPSE tracked changes in global LV function in 9 out of 10 cases, irrespective of RWMA.

**Graphical Abstract:**

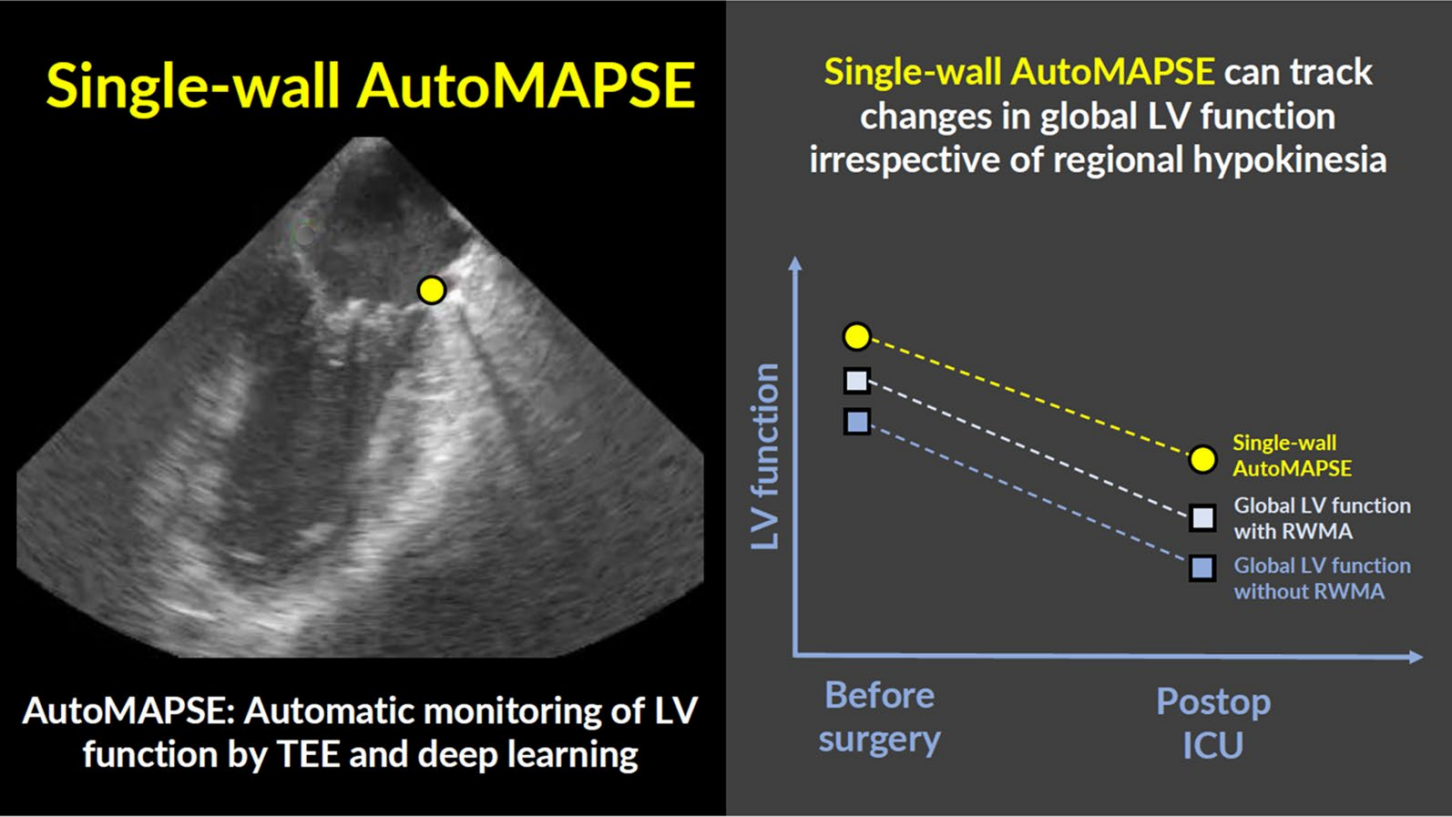

## Introduction

Monitoring global left ventricular (LV) function with transesophageal echocardiography (TEE) during cardiac surgery is an important yet challenging task [1]. Due to competing demands, clinicians usually evaluate LV function by qualitative visual assessment alone. This practice is effective for detecting severe deteriorations in LV function [2]. However, such severe changes tend to be delayed with potentially irreversible complications. A real-time quantitative method that is quick and precise would, therefore, be useful to detect subtler and earlier changes in LV function [3, 4]. This goal could potentially be achieved by measuring the mitral annular plane systolic excursion (MAPSE) in a single wall [5]. MAPSE reflects global LV longitudinal function [3, 6], is more sensitive than left ventricular ejection fraction (LVEF), and can even be measured with TEE without visualizing the entire left ventricle. However, manual echocardiography remains challenging during rapid changes in the patient’s condition and when clinicians face competing tasks.

The potential to improve echocardiographic monitoring has motivated us to develop *autoMAPSE*, a novel method that combines 2D TEE with deep learning to monitor MAPSE automatically [5, 7, 8] and continuously [9]. Several studies have shown that MAPSE decreases after myocardial infarction in all the walls of the left ventricle, even in those remote from the infarcted territory [10–14]. However, by only reporting the average effects, the cited studies [10–14] do not address whether changes in MAPSE measured from a single wall can be used to adequately track changes in global LV function in individual patients with regional wall motion abnormalities (RWMA). This uncertainty must be resolved, not only because RWMA is common in both perioperative and critically ill patients [1, 15–18], but the excellent feasibility for monitoring LV function with autoMAPSE relies on measurements from a single wall [5, 7, 9].

Thus, this study aimed to test the ability of single-wall autoMAPSE in tracking changes in manual global MAPSE (i.e., trending ability) and to explore how this trending ability was affected by RWMA.

## Methods

The present study is a secondary analysis of a patient cohort for which other data have been published previously [8, 9, 19–22]. The inclusion criteria were adult patients scheduled for on-pump cardiac surgery with intraoperative TEE. Exclusion criteria were age less than 18 years, unwillingness to participate, and contraindications to TEE. This study adheres to the principles of the Declaration of Helsinki. Approval was granted by the Regional Committee for Ethics in Medicine (REK number 2022/345556), and all participants provided written informed consent before inclusion. We adhered to the Strengthening the Reporting of Observational Studies in Epidemiology (STROBE) checklist when composing this manuscript [23].

### Echocardiographic image acquisition

We obtained 2D midesophageal two- and four-chamber images of the left ventricle at two time-points: (1) before commencing cardiopulmonary bypass (CPB) and (2) prior to extubation in the postoperative intensive care unit (ICU). The recordings were acquired by retroflecting and turning the probe (6VT-D connected to an E95 scanner, GE Healthcare, Horten, Norway) with the omniplane at 90 degrees to acquire a non-foreshortened midesophageal two-chamber (2C) view. Simultaneous biplane imaging (Multi-D^®^, GE Healthcare, Horten, Norway) was used to minimize LV foreshortening by aligning the secondary sector through the LV apex and rotating the omniplane angle in the secondary sector to approximately 0–30 degrees to obtain an optimized midesophageal four-chamber (4C) view with minimal foreshortening [22]. Finally, the sector width was maximized, and the temporal resolution was increased to at least 40 frames per second.

### Automatic and manual measurement of MAPSE

AutoMAPSE and manual MAPSE were analyzed in the same pre-CPB and postoperative ICU recordings. Their respective changes from pre-CPB to ICU were calculated. MAPSE was measured as the mean of three to five cardiac cycles in the pre-CPB recordings and the mean of up to 10 cycles in the ICU [9].

The technical details and validation of autoMAPSE have been reported previously in detail [7–9]. Briefly, autoMAPSE is based on a deep learning convolutional network trained under supervision to detect the mitral annulus in 2D TEE recordings. The method was trained on recordings obtained from cardiology patients [7, 8] and validated in patients after cardiac surgery [5, 9]. AutoMAPSE detects the mitral annulus in each echocardiography frame, then measures MAPSE as the longitudinal distance traveled by the annulus from end-diastole to end-systole. Here, end-diastole is defined as the mitral annular point closest to the probe timed around the R-wave of the ECG, whereas end-systole is the point of nadir. To avoid erroneous outliers, the method rejects a cardiac cycle if the mitral annulus has a frame-to-frame distance of more than 5 mm, if the annulus is detected in less than 60% of the frames per cycle, or if the highest point does not correspond to the definition of end-diastole. Finally, autoMAPSE reports the MAPSE of each specific wall as the mean of all feasible cardiac cycles in that specific recording. Thus, using autoMAPSE in the 2C and 4C views provides MAPSE in four walls: anterior, inferior, lateral, and septal. AutoMAPSE is defined as feasible when it reports the MAPSE from a single wall, irrespective of the number of cardiac cycles measured [9].

*Manual MAPSE* was measured in B-mode recordings of the same four walls using EchoPAC software version 2.06 (GE Healthcare, Horten, Norway) [9], and the average MAPSE of these walls (global MAPSE) was used to define *global LV function*. We chose this definition of global LV function for two reasons. First, parameters of LV longitudinal function, such as MAPSE, are more sensitive to changes in LV systolic function than LVEF [6]. Indeed, our previous reports in this patient sample show that MAPSE decreases immediately after surgery [9, 22], whereas several studies show that LVEF remains relatively unchanged [19, 22, 24, 25]. Second, speckle-tracking analysis of global longitudinal strain is not possible with simultaneous biplane imaging (Multi-D^®^, GE Healthcare, Horten, Norway) with the present vendor (EchoPAC software version 2.06, GE Healthcare, Horten, Norway). Furthermore, global longitudinal strain requires pristine image quality of the left ventricle in all three standardized views, including the apex, which is generally challenging in the perioperative setting. The feasibility of global MAPSE was defined as the average manual MAPSE of all available walls and did not require measurements from all four.

### Analysis of regional wall motion abnormalities

RWMA was analyzed in the same four walls as MAPSE (anterior, inferior, lateral, and septal) and dichotomously reported as present/absent if any segment of that wall was either hypokinetic, akinetic, or dyskinetic [26]. Two independent observers (JY and OG) assessed all the recordings for RWMA. Cases of disagreement were settled by a third independent observer (EARB) who was blinded to the conclusions of the first two observers. If disagreement was due to one observer rating the image as inadequate and the other rating it adequate, the final decision of image adequacy and RWMA was determined by the third observer, irrespective of the evaluation performed by the first two.

To explore how RWMA affects the ability of single-wall autoMAPSE in tracking global LV function, we first classified whether the RWMA was in the same wall as single-wall autoMAPSE or in a remote wall, where a remote wall was defined as any wall other than the one monitored by autoMAPSE. Next, we classified the evolution of those RWMA to be either persistent or dynamic, where dynamic RWMA comprised either newly developed RWMA postoperatively or the resolution of a pre-existing one. These two definitions yielded four RWMA–autoMAPSE patterns for analysis: (i) remote wall, dynamic RWMA, (ii) remote wall, persistent RWMA, (iii) same wall, dynamic RWMA, and (iv) same wall, persistent RWMA.

### Statistical analysis

The primary objective of the present study was the trending ability of single-wall autoMAPSE as compared to manual global MAPSE. We analyzed trending ability using the four-quadrant plot and reported the primary outcome as the concordance rate with an exclusion zone [27]. Here, a concordant change means that autoMAPSE and manual global MAPSE change in the same direction, irrespective of magnitude. In this analysis, the exclusion zone was defined as the *least significant change* of autoMAPSE by hands-on imaging (1.8 mm), reported previously [9]. In general, a concordance rate of 90% is considered adequate [28, 29]. The secondary objective was to explore the impact of the four RWMA–autoMAPSE patterns on the trending ability of single-wall autoMAPSE.

The absolute change in autoMAPSE and manual global MAPSE was compared using paired *t* tests. Agreement for RWMA analysis between the initial two observers was assessed using Cohen’s kappa. Data are reported as the mean ± standard deviation or median [25–75 percentiles]. The statistical analysis plan was established prior to seeing the results. Missing data were not replaced. As the present study is a secondary analysis, the sample size was fixed to that of the primary study [9]. Statistical significance was defined as a *P* < 0.05. Stata 19.5 (StataCorp LLC) was used for all analyses.

## Results

Fifty one patients were enrolled, of whom 49 remained for final analysis (Table 1). One patient was excluded from the ICU due to an airway issue unrelated to TEE, and another patient had missing pre-CPB recordings. The feasibility of autoMAPSE in the present study was 97% in the anterior wall, 84% in the inferior wall, 89% in the lateral wall, and 92% in the septal wall. The feasibility of manual global MAPSE was 100%, where 75% of all measurements comprised all four walls, 87% at least three, and 100% comprised at least two walls, averaging 3.6 ± 0.7 walls per measurement of manual global MAPSE.

**Table 1:** Patient characteristics

Variables	Values (*N* = 49)
Age (years)	67 ± 8
Male/female (*n*, %)	38/11 (78/22)
Height (cm)	175 ± 8
Weight (kg)	83 ± 16
BMI (kg/m^2^)	27 ± 5
**Coronary artery disease **(*n*, %)	**30 (61)**
Left main stenosis	9 (18)
1-vessel disease	5 (10)
2-vessel disease	8 (16)
3-vessel disease	17 (35)
Recent myocardial infarction (*n*, %)	14 (29)
Preoperative troponin-T (ng/L)	16 [11–51]
Preoperative NT-pro BNP (ng/L)	481 [141–1342]
**Preoperative LVEF** (*n*, %)	
> 50%	35 (71)
31–50%	10 (20)
21–30%	4 (8)
Preoperative RWMA (*n*, %)	17 (35)
**MAPSE pre-CPB (mm)**	
Anterior autoMAPSE	8.7 ± 2.7
Inferior autoMAPSE	11.2 ± 2.9
Lateral autoMAPSE	11.3 ± 3.0
Septal autoMAPSE	8.1 ± 2.0
Manual global MAPSE	11.1 ± 2.7
**MAPSE postoperative ICU (mm)**	
Anterior autoMAPSE	6.7 ± 2.0
Inferior autoMAPSE	7.5 ± 2.6
Lateral autoMAPSE	8.1 ± 2.4
Septal autoMAPSE	6.4 ± 1.9
Manual global MAPSE	7.6 ± 2.1
**Operation **(*n*, %)	
Isolated CABG	23 (47)
Aortic valve replacement	20 (41)
Mitral valve replacement	4 (8)
Mitral valve repair	1 (2)
Surgery on the thoracic aorta	6 (12)
Redo cardiac surgery	3 (6)
EuroScoreII (%)	1.6 [1.0–2.66]
Aortic cross-clamp time (min)	72 ± 35
Cardiopulmonary bypass time (min)	98 ± 46

Bold signifies a category with subgroups

Values are mean ± SD, *n* (%), or median [interquartile range]

*AutoMAPSE* automatic measurements of mitral annular plane systolic excursion in a single wall using deep learning and transesophageal echocardiography, *BMI* body mass index, *CABG* coronary artery bypass grafting, *CPB* cardiopulmonary bypass, *LVEF* left ventricular ejection fraction, *MAPSE* mitral annular plane systolic excursion measured manually on hands-on images, *NT-pro BNP* N-terminal pro B-type natriuretic peptide, *RWMA* regional wall motion abnormality

### Trending ability of single-wall autoMAPSE

**Fig. 1:**
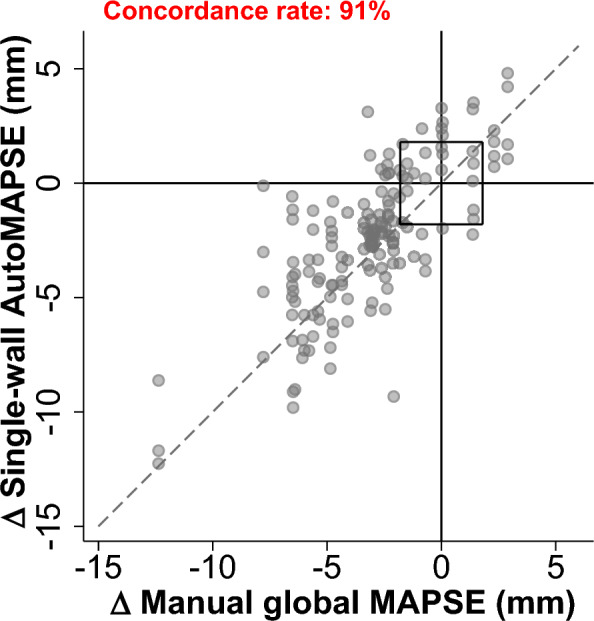
Four-quadrant plot showing the pooled trending ability of single-wall autoMAPSE versus manual global MAPSE (*n* = 138 paired observations). The origin box indicates a central exclusion zone of 1.8 mm, which corresponds to the *least significant change* of autoMAPSE. The dashed line shows the identity line. Single-wall *Single-wall Auto* automatic measurements of mitral annular plane systolic excursion in a single wall using deep learning and transesophageal echocardiography, *MAPSE* mitral annular plane systolic excursion

**Fig. 2:**
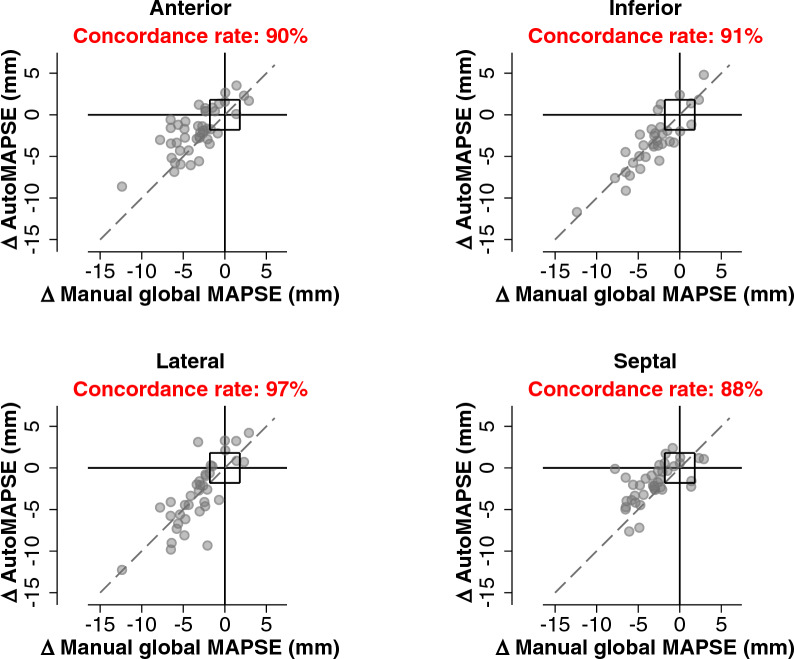
Four-quadrant plots showing the trending ability of single-wall autoMAPSE for each of the four left ventricular walls. The origin box indicates a central exclusion zone of 1.8 mm, which corresponds to the *least significant change* of autoMAPSE. The dashed line shows the identity line. After applying the exclusion zone, the number of paired observations for each wall was 39 for the anterior wall, 32 for the inferior wall, 34 for the lateral wall, and 33 for the septal wall. *AutoMAPSE* automatic measurements of mitral annular plane systolic excursion in a single wall using deep learning and transesophageal echocardiography, *MAPSE* mitral annular plane systolic excursion

Overall, single-wall autoMAPSE had adequate trending ability in all but 12 of 138 cases, with a concordance rate of 91% (Fig. 1). None of the 12 failed cases showed signs of intraventricular dyssynchrony or a mobile apex, nor did they have mitral valve prosthesis. Trending ability for each wall was also adequate except for the septal wall, which failed to meet the adequacy threshold by 2% (Fig. 2).

Single-wall autoMAPSE decreased significantly in all four walls from pre-CPB to the ICU (anterior −2.0 mm, inferior −3.6 mm, lateral −3.1 mm, and septal −1.8 mm; *P* < 0.001 for all), as did manual global MAPSE (−3.5 mm, *P* < 0.001). The absolute changes in single-wall autoMAPSE differed significantly from those of global MAPSE in the anterior and septal wall (Table 2), suggesting a lack of interchangeability between these two parameters.

**Table 2:** Changes in single-wall autoMAPSE compared to paired changes in manual global MAPSE

AutoMAPSE (wall)	Observations (*n*)	ΔAutoMAPSE (mm)	ΔManual global MAPSE (mm)	Difference (mm)	*P*-value
Anterior	45	−1.9 ± 2.7	−3.3 ± 2.8	1.4 ± 2.0	< 0.001
Inferior	34	−3.2 ± 3.4	−3.1 ± 3.1	−0.1 ± 1.7	0.71
Lateral	37	−3.1 ± 4.0	−3.2 ± 3.0	0.1 ± 2.4	0.88
Septal	39	−1.8 ± 2.3	−3.0 ± 2.6	1.22 ± 2.1	< 0.001

The values for *ΔManual global MAPSE* vary for each row, because they show four paired comparisons with each wall. Values are mean ± SD

*AutoMAPSE* automatic measurements of mitral annular plane systolic excursion in a single wall using deep learning and transesophageal echocardiography, *MAPSE* mitral annular plane systolic excursion

Notably, the overall trending ability for manual single-wall *MAPSE* was also adequate (concordance rate 95%, pooled from all four walls). A subgroup analysis showed that trending ability was also adequate for each of the four walls with single-wall manual MAPSE, with concordance rates ranging from 93% (lateral wall) to 100% (anterior wall).

### Effects of regional wall motion abnormalities

RWMA was detected in the anterior wall in 27% of the recordings (indeterminate 3%), inferior in 21% (indeterminate 11%), septal in 29% (indeterminate 1%), and lateral in 13% of the recordings (indeterminate 16%). The RWMA analysis had moderate agreement between the initial two observers before the third observer intervened (kappa = 0.59).

**Fig. 3:**
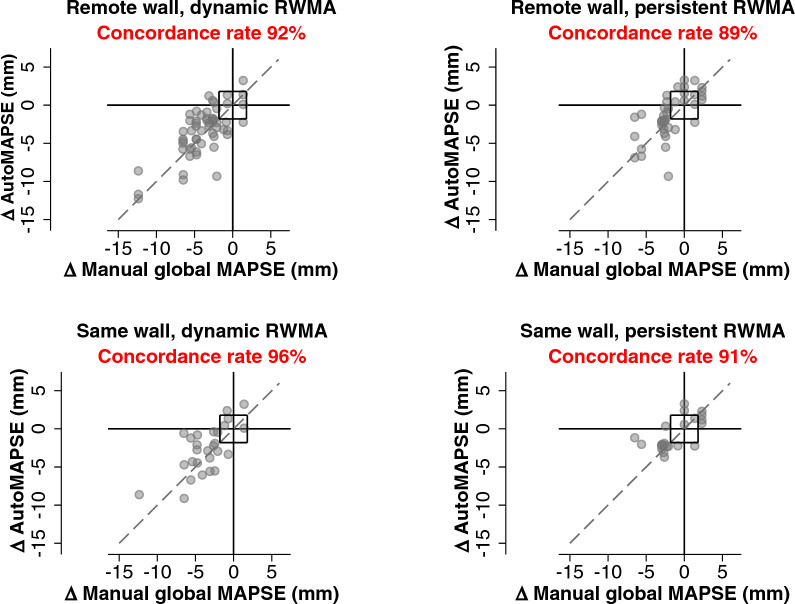
Four-quadrant plots showing the trending ability of single-wall autoMAPSE for the four patterns of regional wall motion abnormality. The origin box indicates a central exclusion zone of 1.8 mm, which corresponds to the *least significant change* of autoMAPSE. The dashed line shows the identity line. *AutoMAPSE* automatic measurements of mitral annular plane systolic excursion in a single wall using deep learning and transesophageal echocardiography, *MAPSE* mitral annular plane systolic excursion, *RWMA* regional wall motion abnormality

The effects of the four RWMA patterns on the trending ability of single-wall AutoMAPSE were negligible, as only one of the patterns showed a marginally inadequate concordance rate of 89% (Fig. 3).

## Discussion

Our main finding is that single-wall autoMAPSE tracks changes in global LV function in 9 out of 10 cases. This was also the case when single-wall MAPSE was measured manually. The effect of RWMA on this trending ability was negligible. Thus, it appears that MAPSE from one wall, measured manually or by autoMAPSE, may track changes in global LV function, irrespective of RWMA.

RWMA was relatively prevalent in this study, but it is doubtful that all these cases represent myocardial ischemia. Not only can myocardial stunning or the use of pacemakers induce RWMA, but postoperative patients may suffer from relative or absolute hypovolemia, which can cause both global and regional hypokinesia [30, 31]. RWMA of any etiology was relevant for the study’s objective, even if they did not necessarily represent myocardial ischemia.

### Why single-wall MAPSE tracks global left ventricular function irrespective of regional wall motion abnormalities

The ability of single-wall MAPSE in tracking global LV function, even during RWMA, may be explained by two complementary mechanisms: first, the geometric relationship, whereby global MAPSE is proportional to stroke volume and inversely proportional to the LV outer cross-sectional area [32], and second, the effects of *tethering,* where all the contractile movements in the left ventricle affect the entire mitral annulus and can be monitored by quantifying MAPSE at a single wall.

An acute RWMA of a certain magnitude reduces LV stroke volume and emptying, which in turn increases LV end-systolic and end-diastolic volumes [33]. This acute ventricular dilatation increases afterload in early-systole according to the Law of Laplace and preload in end-diastole, which may or may not maintain stroke volume depending on the magnitude of the RWMA, the position of the left ventricle on the Frank–Starling curve, and the contractile status of the remaining myocardium. Irrespective of the effects on stroke volume, this LV dilatation will increase the LV outer cross-sectional area and, therefore, reduce the global MAPSE, given the inverse relationship between MAPSE and LV outer cross-sectional area [32]. The effect of RWMA on global MAPSE is, therefore, determined by the balance between LV stroke volume and its cross-sectional area. As the entire annular ring is tethered to the left ventricle, RWMA in one wall will affect the MAPSE in a remote wall, depending on the factors mentioned. The same mechanisms explain why it is still useful to monitor the single-wall MAPSE of a persistently akinetic wall. The akinetic wall is anchored to the mitral annulus and the remaining functional segments. When these segments shorten, traction is applied to the akinetic region, and this motion is translated into the mitral annulus. However, these mechanisms may be less reliable in patients with intraventricular dyssynchrony, mitral valve prosthesis, or an abnormal mobile apex. Although we did not identify these potential limitations in any of the failed cases, they may still be important. Nevertheless, single-wall MAPSE does not reflect regional function, but the global interactions between LV loading conditions and contractility.

### Clinical implications

These findings have some practical implications when using MAPSE to monitor LV function: MAPSE from a single wall is likely adequate for serial monitoring of global LV function. This means that the already-simple method of averaging MAPSE from the septal and lateral walls can be further simplified to one wall for clinicians with competing tasks. When only a single wall is required for MAPSE, the advantage is that the clinician can choose the view with optimal image quality, which can improve the measurement reproducibility and precision during monitoring.

Regarding the use of single-wall autoMAPSE, we should note that the absolute values of MAPSE [5, 9, 19, 34] and their respective changes differ between walls. Thus, the absolute measurement of single-wall MAPSE is not necessarily interchangeable between walls or with the global MAPSE. Furthermore, measurements of MAPSE with B-mode are not interchangeable with M-mode [35]. Thus, we should not blindly apply normal values of MAPSE derived from larger epidemiological studies. AutoMAPSE in the anterior wall has repeatedly been the most feasible one [5, 9], whereas this present study suggests that the changes in lateral wall autoMAPSE are most interchangeable with the changes in manual global MAPSE. Finally, in line with previous studies [10–14], we should not use MAPSE in a wall to detect new-onset RWMA.

### Limitations

The first limitation is that the sample size was relatively small. Thus, the study may have been underpowered to exclude an effect of RWMA on the trending ability of single-wall autoMAPSE. However, the concordance rates in the four patterns of RWMA suggest that any undetected effect of RWMA on trending ability is relatively small. Second, the septal wall autoMAPSE did not show adequate trending ability (concordance rate 88%). However, a single observation in the other direction would have tipped the results to meet the threshold due to the small sample size. Notably, the same septal wall had adequate trending ability when assessed by single-wall manual MAPSE. It may, therefore, be possible that the septal wall autoMAPSE can be used if no other walls are visible. Nevertheless, the findings of this study, together with previous studies suggesting limited monitoring feasibility in this wall [7, 9], suggest that the septal wall MAPSE should not be the wall-of-choice, especially in patients undergoing cardiac surgery. Third, our data did not allow comparison of changes in MAPSE versus those of global longitudinal strain. However, the two parameters are highly correlated [36], and MAPSE was even found to have superior prognostic abilities compared to global longitudinal strain in a head-to-head comparison using cardiac magnetic resonance imaging in a cardiology cohort [37]. Finally, the subjective visual analysis of RWMA was performed by two echocardiographic researchers and not by expert cardiologists. However, visual analysis has suboptimal reproducibility even among experts [38, 39]. There are currently no gold standards for RWMA analysis, and the method used in this study is the current clinical standard in perioperative and critical care echocardiography. We did not use ultrasound contrast agents to optimize RWMA assessment, as the present study is a secondary analysis of already-obtained data. Novel methods for more accurate quantification of regional function are underway, but none have yet reached clinical application [21, 40]. Given the uncertainty of the assessment of RWMA and the lack of a gold standard, our approach using a third independent observer to settle the disagreement between the first two yielded a consistent agreement among at least two observers.

## Conclusion

In this secondary analysis, MAPSE from a single wall tracked changes in global LV function in 9 out of 10 cases, irrespective of RWMA. Single-wall autoMAPSE has the advantage of being fully automated, quick, and precise, and is, therefore, a feasible tool for automatic monitoring of LV function in patients undergoing cardiac surgery.

## Take-home message

Measuring mitral annular plane systolic excursion (MAPSE) in a single wall is a potentially effective method for monitoring global left ventricular function, especially when automated with a novel deep learning method using transesophageal echocardiography, called *autoMAPSE*. This study found that MAPSE from a single wall, measured manually or by autoMAPSE, adequately tracks changes in global left ventricular function in 9 out of 10 cases, irrespective of regional wall motion abnormalities.

## Data Availability

The datasets used and analyzed during the current study are unavailable to other parties due to a lack of patient consent for this purpose.

## Abbreviations

2C: Midesophageal two-chamber view
4C: Midesophageal four-chamber view
AutoMAPSE: Automatic measurement of mitral annular plane systolic excursion
CPB: Cardiopulmonary bypass
ICU: Intensive care unit
LV: Left ventricular
LVEF: Left ventricular ejection fraction
MAPSE: Mitral annular plane systolic excursion
TEE: Transesophageal echocardiography
RWMA: Regional wall motion abnormality
